# Genetic, Socioecological, and Health Research on Extreme Longevity in Semisupercentenarians and Supercentenarians: A Scoping Review

**DOI:** 10.1155/jare/1605361

**Published:** 2026-04-17

**Authors:** Omnia Abdelraheem, Wafa Abu El Kheir-Mataria, Sungsoo Chun

**Affiliations:** ^1^ Institute of Global Health and Human Ecology, The American University in Cairo, New Cairo, 11835, Egypt, aucegypt.edu

**Keywords:** aging research, extreme longevity, global cohort, methodology, semisupercentenarians, supercentenarians

## Abstract

**Objective:**

People with extreme longevity represent a unique model to study the biology of aging and discover clues to improve the general population’s health. Current scoping reviews systematically map and synthesize the existing literature on supercentenarians and semisuper centenarians to identify key themes, methodologies, and findings that can inform the development of a comprehensive framework for a Global Supercentenarian Cohort Study protocol.

**Methods:**

A scoping review was conducted as per PRISMA‐ScR guidelines using the PEO framework (population: semisupercentenarians aged 105+; exposure: genetics, socioecological, and other factors; outcome: supercentenarians aged 110+). Seven databases, Scopus, ProQuest, PubMed, PsycINFO, Scopus Secondary literature, Scopus Patents, and Cochrane Database of Systematic Reviews (CDSR), were searched, incorporating data from Scopus Secondary literature. Unpublished data were excluded. The scoping review protocol was published to ensure transparency and rigor.

**Results and Discussion:**

144 were included. A thematic analysis of individual studies′ findings was conducted to identify multidimensional themes of supercentenarian health. The following themes were identified: Age validation theme, demographics theme, behavior theme, personality, traits theme, quality of life theme, well‐being and life satisfaction, cognition theme, social theme, religiosity/spirituality theme, independence/self‐ability theme, mental health theme, health theme, genetics theme, and ecological theme. There was variability in the methods of assessment and tools used for each theme across different studies and inconsistencies in terminology, with similar concepts such as social support, social engagement, social contact, and social isolation being referred to by different names in various studies.

**Conclusion:**

We propose a comprehensive framework comprising three components: cohort setting, measuring contributing factors, and full assessment. This framework sets the stage for a unified, supranational protocol that harmonizes survey methods across countries, integrates multidisciplinary perspectives, and adopts a longitudinal approach. It would enable larger sample sizes and more robust statistical analyses, allowing researchers to explore complex relationships and derive more accurate conclusions about supercentenarians′ health, well‐being, and longevity. Developing this unified protocol necessitates the consensus of global experts.

**Highlights**
• Scoping review maps research on supercentenarians and semisupercentenarians.
• Identified 144 studies covering genetics, behavior, social, health, and ecological themes.
• Found inconsistencies in terminology and assessment methods across studies.
• Proposed a unified framework for a global supercentenarian cohort study protocol.
• Framework enables cross‐country harmonization and robust longitudinal analysis.

## 1. Introduction

Limiting illness, both in duration and the number of individuals affected, becomes more critical as human longevity increases. Studying healthy humans with exceptional lifespans is meaningful in discovering clues to improve the general population’s health. Centenarians (individuals aged 100 years or older), semisupercentenarians (aged 105–109), and supercentenarians (individuals aged 110 years or older) are excellent models for the study of healthy longevity. According to research, these aged population surprisingly maintain extraordinary cognitive and physical performance [1].

Current systematic reviews investigate exceptional lifespans from diverse perspectives. They cover a range of themes, each contributing unique insights into the understanding of supercentenarian aging. Some reviews discuss longevity from a biological perspective, such as genetic factors [1–4], microbiome [5], and Inflammation [6, 7]. Others explore the behavioral factors such as diet [8, 9], habits, and lifestyle [10–12]. Cognitive factors are the focus of specific reviews [13], while others discuss psychological aspects such as depression and anxiety [14].

While individual reviews have provided valuable insights into various aspects of supercentenarian aging, the need for more supercentenarian cases and the limitation of studies to mostly developed countries pose challenges. So, we are losing opportunities for valuable cases to investigate globally. There are many unexplored variables, such as geographical variables and interactions between genetics and environment. Recognizing these gaps, there is a need for a holistic and comprehensive approach to investigate this exceptional demographic.

Thus, this scoping review aims to synthesize existing knowledge on supercentenarian aging from diverse perspectives, focusing on a global and comprehensive approach. This approach aims to be all‐encompassing to comprehensively understand the multifaceted aspects of extreme longevity. The objective of this scoping review is to systematically map and synthesize the existing literature on supercentenarians and semisuper centenarians to identify key themes, methodologies, and findings that can inform the development of a comprehensive framework for a Global Supercentenarian Cohort Study protocol. The methodology for this scoping review was previously described in a published protocol [15].

## 2. Methods

### 2.1. Design

This scoping review follows the population, exposure, and outcome (PEO) framework and adheres to the Preferred Reporting Items for Systematic Review and Meta‐Analysis (PRISMA) extension for scoping reviews 2018 guidelines [16]. The scoping review protocol was published to ensure transparency and rigor [15].

### 2.2. Search Strategy

Seven databases were searched from their inception up to 2nd October 2024, with language restrictions. Only peer‐reviewed studies in English were included. The databases were Scopus, ProQuest, PubMed, PsycINFO, Scopus Secondary literature, Scopus Patents, and Cochrane Database of Systematic Reviews (CDSR). A search strategy was created for Scopus and then modified to be specific to each database. The search strategy was developed by exploring the Scopus keyword filter and developing queries by examining relevant terms used in existing systematic reviews. This search strategy was tested and refined to claim it was the most effective strategy for this review. The complete search strategy is described in Supporting File (Appendix A). We reviewed the reference list of other reviews and related articles.

We applied the PEO model to define the research question. The inclusion criteria were as follows:

• Population: Semisupercentenarian (aged 105+) and over.
• Exposure: Genetic, lifestyle, sociocultural, and environment−ecological variance.
• Outcome: Supercentenarian (aged 110+).

Eligibility criteria included peer‐reviewed research articles, both quantitative and qualitative, exploring aspects of semisupercentenarians or supercentenarians. It included observational studies (cohort, case‐control), intervention studies, case reports, or case series. We did not retrieve or include any unpublished data. No restrictions on study settings or publication date were imposed. Studies primarily focusing on individuals under 105 years were excluded.

### 2.3. Study Selection and Data Extraction

Citations and brief records (RIS files) identified by the search strategy were imported electronically to Zotero, and duplicates were removed via Zotero. The number of studies after merging duplicates (138) via Zotero is 706. Then, the titles, keywords, and abstracts of the studies obtained by the search strategy were screened to determine if they met the inclusion criteria. When the inclusion or exclusion of a study could not be based on the screening of the title, keywords, and abstract, in the final stage, the entire article was retrieved and checked for inclusion.

### 2.4. Thematic Analysis

Thematic analysis follows Braun and Clarke’s (2006) six‐phase framework [17], ensuring a structured and rigorous synthesis of qualitative findings from centenarian studies. The process begins with familiarization with data, where researchers review and summarize each study’s findings, noting recurrent themes across different sources. Following this, initial codes are generated inductively, allowing themes to emerge directly from the data. These codes focus on key aspects of centenarian studies, including age validation methods, demographics, health, functional capacity, cognition, mental health, behaviors, social support and relations, quality of life (QoL), personality traits, environmental and genetic factors, and religiosity. Once the initial codes are established, the process moves to searching for themes, where codes are collated into broader themes and subthemes, identifying commonalities and differences across studies. The next step, reviewing themes, involves iterative refinement to ensure consistency, resolving discrepancies through discussion among researchers. Subsequently, defining and naming themes entails assigning concise and descriptive labels to each theme, ensuring theoretical linkages to existing gerontological frameworks.

## 3. Results

### 3.1. Study Selection and Characteristics

**FIGURE 1:**
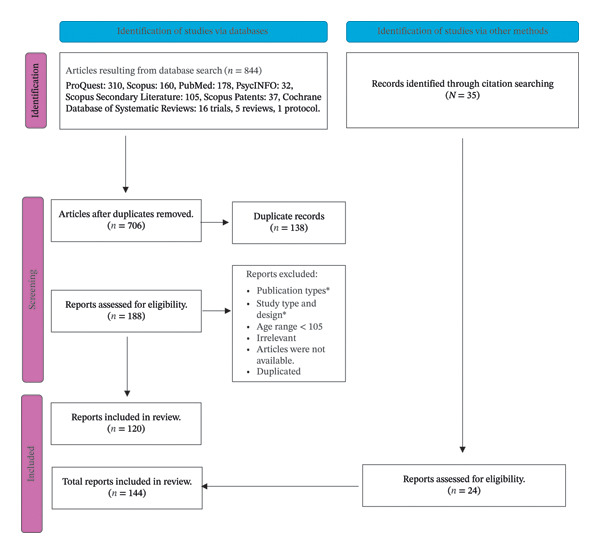
The PRISMA flow diagram presenting the study selection process. ^∗^Publication Types like Systematic review Protocol, Review article, Dissertation/ [unpublished manuscript], Open peer commentary, Annual review Comment/Reply, Book Chapter, Newsletter, Thesis Book, Workshop on Supercentenarians, Editorial Short editorial review, Letter to editor, Research Letter, Retracted Article, Protocol, Systematic Review, News and Views. ^∗^Study Type and Design like Animal models, Molecular and cellular experiments, Analysis of co‐written autobiographies of centenarians, Mortality patterns among siblings of centenarians and siblings of shorter‐lived individuals, Cell line.

From all databases combined, we identified 844 titles: 310 in ProQuest, 160 in Scopus, 178 in PubMed, 32 in PsycINFO, 105 in Scopus secondary literature, 37 in Scopus Patents, and in CDSR, we found 16 trials, 5 reviews, and one protocol. After the exclusion of duplicates, we reviewed a total of 706 studies. Figure 1 shows a flow diagram of the reviewed studies. We excluded studies that did not meet the inclusion criteria defined by the PEO strategy. After screening for eligibility, the abstract resulted in the selection of 188 articles. All those studies were available as full‐text articles. These retrieved articles were subsequently evaluated further according to the inclusion criteria. After screening the full text of each article, 135 studies were included. The manual search resulted in finding other relevant articles for the study and 27 centenarian studies from different geographical locations (Supporting File [Appendix B]). The total included studies are 162 (Figure 1).

### 3.2. Thematic Mapping and Synthesis

#### 3.2.1. Age Validation Theme

Age validation is an important topic in studying the aging population, especially if the research studies cases at the far end of the human life span, starting from 100 years old and above. Studies on this demographic often aim to identify the reasons for living to this extreme age, making actual age verification an essential step in the study.

Age validation was a major cross‐cutting theme in our study. The included studies addressed age validation in three main ways.

First, several studies recruited participants through established cohorts or databases that apply recognized age‐verification procedures, such as Georgia Centenarians Study [18], the Sydney Memory and Aging Study [19] the Semisupercentenarians Study in Japan [1, 20], Fordham Centenarian Study [21, 22], the International Database on Longevity [23–26], Danish Birth Cohort Studies [27], International HapMap project [28], Center d’Etude du Polymophisme Humain (CEPH) Aging cohort [29, 30] New England Centenarian Study [31], Oklahoma 100 Year Life Oral History Project [32], China Hainan Centenarian Cohort Study [33–37], and multiple cohort studies [38–41].

Second, a subset of studies focused specifically on validating supercentenarian ages; many of these were conducted by the Gerontology Research Group (GRG) [42–48].

Third, some studies explicitly reported the validation parameters or eligibility criteria used to confirm age as part of their study methods [10, 40–47].

Age validation is particularly critical in supercentenarian research because months, and sometimes days, can be analytically meaningful when characterizing exceptional survival. Accordingly, the most common approach involved triangulating age across multiple independent records. For example, the GRG validates cases by linking early‐life census records, mid‐life social security documentation, and late‐life sources such as National Death Index records [49].

Across studies, validation commonly drew on early‐life documents (e.g., birth certificates, baptismal records, and family bible entries), mid‐life documents (e.g., marriage certificates, military records, and census data), and late‐life documents (e.g., death certificates, obituaries, and late‐life census records). Additional supporting sources included population registers, family books, national identification cards, and passports [50]. Some studies also reported complementary verification strategies such as family‐tree reconstruction, third‐party confirmation, consideration of local community context [51], and linkage to national health insurance databases [52].

Taken together, all included studies either relied on cohorts/databases with established age validation protocols or specified their own explicit criteria for confirming participant age.

#### 3.2.2. Demographics Theme

The second theme in our study is the demographics of the supercentenarian population included in each study. Demographics helps in understanding extreme longevity, which provides essential context for supercentenarian studies. Usually collected demographics include age, gender, geographic location, ethnicity, and socioeconomic factors. In our research, studies can roughly be grouped into three categories: studies that did not elaborate on the demographics of their subjects. These are mainly studies that were performed using supercentenarian data obtained from famous cohort studies [18, 22–25, 27, 29–40, 42–47, 53–75]. The other groups are studies that mentioned the demographics collected on their subjects [76–90]. Finally, the rest of the studies did not mention the demographics. The most common demographics collected were name, date of birth, place of birth, residence, race, sex, and age. However, some studies collected more extensive data including name, date of birth, date of death, completed age in YYYDDD (calculated), place of birth, place of residence (last), marital status (last), citizenship (last), year of birth, presence/absence in the database t, t+1, and all the information about migration from/to another municipality [91]. Others included information such as maximum reported age at death (MRAD), hazard rate [41], and education level [18, 35, 55, 59, 74].

Demographics in supercentenarian studies are as important as in any other type of study investigating a special phenomenon. Demographics provide prevalence insights into which populations of supercentenarians are present. They also help identify geographic clusters of supercentenarians, which aids in pinpointing potential genetic or environmental factors and health disparities in longevity. According to the included studies, the populations with the highest number of supercentenarians are in Japan, China, Italy, the United States, and Sicily. Other countries, such as Iran, Cuba, and France, were present in some studies but to a lesser extent than the previously mentioned countries. Thus, demographics could assist in directing future research and policymaking in the areas of geriatrics and longevity.

#### 3.2.3. Behavior Theme

Past and current behavioral attributes of supercentenarians are part of supercentenarian studies. In our research, fourteen out of the included studies collected information about one or more of their subjects’ behaviors and habits. The most common behavioral domain among these studies was smoking. Ten studies asked about the supercentenarian smoking status (never smoked, past smoker, current smoker, and passive smoking) [34, 35, 52, 71, 80, 90, 92–95]. The second domain was alcohol consumption, where nine studies asked about alcohol habits (never drank, past drinker, and current drinker) [34, 35, 62, 70, 71, 87, 90, 93–96]. The third domain was diet. Five included studies reported information concerning their subjects′ diet and nutrition habits, such as meal frequency [94], dietary habits and types of foods mostly consumed [80], nutritional status using MNA‐SF [72, 97], and caloric intake [98]. The fourth domain was sleep mode, quality, and duration, with four included studies [35, 80, 90, 94]. The fifth domain was physical activity and exercise [34, 90], followed by tea drinking [87, 94], dental hygiene [71], and sexual life [94].

#### 3.2.4. Personality Traits Theme

Personality traits influence an individual’s behavior and relationships, which in turn affect health and, consequently, longevity [97]. Despite their recognized role in longevity, personality traits have received limited attention in supercentenarian research. Among the studies included in our review, only one examined personality traits as a potential factor in extreme longevity. This study used the 10‐item version of the Big Five Inventory (BFI) to assess personality traits and explored their connection to social isolation and loneliness among participants [21].

Personality traits refer to enduring patterns of thinking, feeling, and behaving, typically classified into five main dimensions: extraversion (sociability and outgoingness), agreeableness (cooperativeness, compassion, and consideration), conscientiousness (organization, responsibility, and goal orientation), emotional stability (resilience and steadiness), and openness to experience (curiosity and creativity). While personality traits have been discussed in longevity research [52, 99–101], their measurement in supercentenarians remains scarce. This may be because these traits overlap with other themes in our study, such as behavior and social dimensions, leading researchers to integrate them within broader analyses rather than study them as separate factors.

#### 3.2.5. QoL Theme, Well‐being, and Life Satisfaction

In our research, nine studies covered these areas. Three studies assessed well‐being using one of the following: the Philadelphia Geriatric Center Moral Scale (PGCMS) [52, 99–101], the 5‐item WHO (WHO‐5) Well‐Being Index [40], and the Diener Satisfaction of Life Scale [75]. Three studies assessed the QoL using one of the following: the Short Form‐12 (SF‐12), which is a concise questionnaire with 12 items that assess various dimensions of health‐related QoL [99], and EuroQol 5 Dimension (EQ‐5D), which is a standardized instrument for assessing health‐related QoL through measuring five domains: mobility, self‐care, usual activities, pain/discomfort, and anxiety/depression [33, 94]. Three studies assessed life satisfaction using a 5‐item satisfaction life scale [21, 22] and QoL scoring [102].

#### 3.2.6. Cognition Theme

Cognition was an important theme in our research; 24 included studies assessed cognitive functions in their extremely aged subjects. Different methods were used to assess cognitions in these subjects. The most common instrument was the Mini‐Mental State Examination (MMSE), which was applied in 20 studies [18, 21, 27, 35, 40, 52, 52, 55, 75, 80, 83, 87, 90, 94, 95, 98, 100, 103, 104]. Variants such as the Italian version of the MMSE [99] and the MMSE‐2 Brief Version [55] were also used. Some included studies used other cognitive assessments, namely, the Japanese version of the Montreal Cognitive Assessment (MoCA‐J) [101], Blessed Information−Memory−Concentration (BIMC) Test [105], and the Global Deterioration Scale (GDS) [21, 64]. Finally, some studies combined more than one test [52, 59, 83, 104].

#### 3.2.7. Social Theme

Nine of the included studies discussed various social subthemes among supercentenarians [18, 21, 22, 29, 63, 94, 97, 102, 106]. Some studies measured the same subtheme but used different terminology. For example, the number of children was assessed in two different studies: one as a social indicator [22] and the other as a social resource [21]. We grouped the same measures and harmonized the terminology, resulting in the identification of the following subthemes: Social support was assessed using the 24‐item Social Provisions Scale [94]. Social network (isolation) was measured with the 6‐item Lubben Social Network Scale (LSNS‐6) [21, 22]. Under the social resources’ subtheme, we included widowhood status and duration, living situation, having living children, the number of living children, having living grandchildren, the number of children living close by, and subsidies, which include financial assistance or aid provided by family members, friends, or social programs [22].

One study assessed the social engagement subtheme (past and current) via proxies [18]. They followed a selection procedure where, if spouses were alive, they were first selected as proxies. Second, adult children were contacted to provide proxy reports. For participants who had more than one living child, proxies were nominated by the participants. The third possible group of proxies included other family members, and the final group included community members. Because proxies could have been in advanced age, all proxies were tested for cognitive functioning (i.e., MMSE), and cognitively impaired proxies were excluded from the study. Proxies provided information on the number of times in the past week the supercentenarians had spent time with someone. This measure, based on Fillenbaum’s work (1988), assessed current social engagement. They also asked proxies whether the centenarians or octogenarians had ever been engaged in a series of cognitive engagement tasks [18] to measure the past engaged lifestyle.

#### 3.2.8. Religiosity/Spirituality Theme

Only one study assessed this theme, asking participants, “Are you religious?” The response options were as follows: (1) Yes, I go to church, or I pray every day, (2) yes, I watch church services on TV on Sunday, (3) I’m a believer but not practicing, and (4) I’m not a believer [90].

#### 3.2.9. Independence/Self‐Ability Theme

Thirty studies covered this theme, and four subthemes were identified: functional status, fatigue, pain, and frailty. For functional status, contributing studies differ in their assessment of this subtheme, both in the instruments used and in the areas of functional status assessed. All the tools used in the studies were extracted from the area that they assess. Seven studies assessed the Basic Activities of Daily Living (BADL) assessment with the Barthel Index. The index was used to capture the functional independence of individuals in performing essential self‐care tasks [52, 79, 92, 95, 99, 103, 105]. One study used the Katz Index of Independence in Activities of Daily Living (ADL) for assessing individuals′ level of independence in basic self‐care tasks [98]. The modification of the questionnaire developed by Verghese et al. was found suitable for assessing Advanced Activities of Daily Living (AADL), particularly in investigating participation in leisure activities among older adults [103]. The Lawton scale (Lawton−Instrumental Activities of Daily Living [IADL]) adequately assessed the IADL, reflecting individuals′ ability to maintain independence in tasks essential for living independently [37, 40, 72, 103]. Finally, the Older Americans Resources and Services (OARS) Multidimensional Functional Assessment Questionnaire was used for measuring functional status across various domains among older adults [21, 75, 103].

For the fatigue subtheme, two studies [18, 103] used the Multidimensional Fatigue Inventory (MFI). MFI is a 20‐item self‐report instrument designed to measure fatigue. It covers the following dimensions: General fatigue, physical fatigue, mental fatigue, reduced motivation, and reduced activity. One study used The Fatigue Severity Scale [98].

For the pain subtheme, two studies assessed the severity of physical pain using the numerical rating scale [22, 95].

Although fatigue and frailty are interconnected in older adults, separate assessment tools for frailty were extracted from the studies included in our systematic review, and it is considered as a separate subtheme. Two studies [71, 103] assessed the frailty phenotype, which is based on a predefined set of five criteria exploring the presence/absence of signs or symptoms (i.e., involuntary weight loss, exhaustion, slow gait speed, poor handgrip strength, and sedentary behavior) using self‐reported data. In this study [81], the frailty index, which is measured using 40 items that represented deficits in a range of systems, including comorbidities, activities of daily living, physical tasks, cognition, and performance testing, was used to assess the frailty [81]. One study assessed the unintentional weight loss without explicitly stating that it is related to frailty [94].

#### 3.2.10. Mental Health Theme

Fourteen studies in our review assessed different aspects of mental health, which we categorized into five subthemes. Anxiety was assessed using the Beck Anxiety Inventory in one study [99]. Depression was evaluated in nine studies using the Geriatric Depression Scale [21, 22, 75, 80, 90, 94, 99, 107]. Coping was measured with the Multidimensional Coping Inventory in one study [75], while psychological strengths, including personality dimensions such as extraversion, neuroticism, agreeableness, openness, and conscientiousness, were evaluated with the BFI in another study [75]. Loneliness was assessed using the UCLA Loneliness Scale in two studies [22, 97]. Finally, mental well‐being, and in another study referred to as psychological frailty, was examined using the WHO‐5 Well‐Being Index [101, 103].

#### 3.2.11. Health Theme

Most of the included studies assessed the health of the supercentenarians. The data and tools collected from the included studies provided a comprehensive health assessment of supercentenarians, and several key subthemes were identified. The mortality and multimorbidity analysis subtheme addresses overall mortality rates, the presence of multiple chronic conditions, and the number of diseases, incorporating survival analysis and self‐rated health assessments [21, 27, 34, 40, 52, 55, 99]. The Cumulative Illness Rating Scale−Geriatric was used to assess and quantify the burden of disease in elderly patients [99].

The health conditions and past medical history theme includes a range of health conditions, including cardiovascular health, reproductive history, cerebrovascular history, and various other conditions such as arthritis, infectious diseases, respiratory and kidney conditions, gastrointestinal issues, diabetes, vision or hearing problems, cancer, and dementia [20–22, 29, 33, 36, 40, 52, 64, 68, 72, 75, 79, 80, 87, 90, 92, 94, 95, 98, 99, 102–105, 108–111].

The clinical examination subtheme includes anthropometric measurements, physical examinations, and specific assessments such as feces examination, electrocardiogram (ECG), and ultrasonography. It also covers dental and gynecological check‐ups. The hematology and biochemistry subtheme focuses on various blood and biochemical markers, including blood counts, glucose levels, proteins, lipids, and other biochemical parameters [29, 34, 40, 52, 52, 54, 60, 80, 86, 90, 92, 94, 95, 98, 99, 103, 109, 112–115].

Finally, the medication subtheme documents current medications, the presence of polypharmacy, and the Anticholinergic Drug Scale (ADS) score [29, 52, 68, 87, 99, 100, 104], while the health service use subtheme captures data on outpatient attendance, emergency treatments, hospitalizations, and health insurance coverage [52, 94, 99]. These subthemes collectively provide a comprehensive framework for assessing the health status and medical history of supercentenarians, contributing valuable data for systematic reviews and studies focused on aging and longevity.

#### 3.2.12. Genetics Theme

In supercentenarian studies, the identified themes delve into the genetic and epigenetic factors contributing to exceptional longevity. Genomic variants and gene expression patterns, including single‐nucleotide polymorphisms (SNPs) [39, 40, 61, 69, 80, 109, 116], and mitochondrial DNA (mtDNA) variants [78], [117] play a crucial role in understanding the unique genetic makeup of supercentenarians. The study of molecular markers [81] [167], alongside gene expression patterns, provides insights into the biological mechanisms underlying extreme longevity [57], [86], [116], [118], [119]. Epigenetics emerges as a vital theme, exploring how telomere length and maintenance [80], [81], epigenetic modifications, and the association of epigenetic age with mortality rates and cardiovascular disease (CHD) risk factors impact longevity [82]. The epigenetic clock and DNA methylation age (DNAm Age) are key indicators of biological aging [1, 30, 67], while MicroRNAs and blood mononuclear cells (PBMCs) methylation data offer further understanding of gene regulation in supercentenarians. Extrinsic epigenetic age acceleration (EEAA) quantifies deviations from typical aging patterns [80, 90, 119, 120]. Quantitative analysis and risk assessment, through quantitative trait association analysis and polygenic risk scores (PRSs), assess genetic predispositions to longevity [110, 121]. Finally, the role of the microbiome highlights the importance of microbial interactions in the health and lifespan of supercentenarians [87, 122–124] (Table 1).

**TABLE 1:** Genetic variants associated with exceptional longevity.

Subtheme	Element	Evidence
Genomic variants and gene expression	Single‐nucleotide polymorphisms (SNPs): these are variations in a single‐nucleotide base within the DNA sequence that can be used to unlock the genetic determinants of exceptional longevity [39, 40, 61, 69, 80, 109].	• T/G polymorphism at −330 nt of the IL‐2 gene promoter region • rs2802292 G‐allele (G > T) of the Forkhead box O3A (FOXO3A) gene and Apolipoprotein (Apo) E • SOD3 p.R231G (rs1799895 C > G) • Five significant SNPs were identified in two loci: 2p22.1 (inside gene DHX57) and 16p13.3 near gene MLST8 • rs2075650 in TOMM40 • rs405509 in APOE • rs12978931 in PVRL2 • rs519825 in PVRL2 • rs395908 in PVRL2 • rs2075650 in the TOMM40/APOE locus • 18 SNPs in the RNA editing genes ADARB1 and ADARB • SNPs in genes CDH13 and ADIPOQ associated with circulating high‐molecular‐weight (cHMW) adiponectin levels
	Mitochondrial DNA (mtDNA) variants: mitochondrial DNA (mtDNA) is a distinct genetic entity found in the mitochondria of eukaryotic cells, separate from the nuclear genome The mitochondrial genome occurs in multiple copies, resulting in both homoplasmic and heteroplasmic pathogenic mtDNA variants, leading to a dynamic and tissue‐specific mosaic pattern of oxidative deficiency and marked phenotypic variation [78, 117]	• Mitochondrial haplogroups (G2a and N9b1) • M9 haplogroups show a significant reduction in longevity subjects compared to elderly and middle‐aged subjects • The frequency of N9 haplogroups decreases significantly from middle‐aged to elderly to longevity subjects • Gender‐stratified analysis reveals significant trends in D4 and B4a haplogroup frequencies among females, indicating associations with longevity
	Genetic and molecular markers [81]	• Superoxide dismutase 3 (SOD3), an antioxidant enzyme, is known as extracellular SOD (EC‐SOD): EC‐SOD co‐operates with adiponectin and possesses beneficial functions for DM in the oldest‐old • Predicted levels of ◦ Adrenomedullin ◦ Beta‐2‐microglobulin ◦ Cystatin‐C ◦ Growth differentiation factor 15 ◦ leptin ◦ Plasminogen activator inhibitor 1 ◦ Tissue Inhibitor metalloproteinase 1
	Gene expression patterns: Changes in gene expression, or how genes are turned on or off, can influence aging and longevity [57, 57, 86, 116, 118, 119].	• TSHZ3 gene • The SNP in 16p13.3 was found to have a cis‐acting effect on the expression levels of MLST8 in most brain regions • Similarly, the SNP in 2p22.1 had a cis‐effect on DHX57 expression levels in cerebellar samples • The study identifies 27 metabolic variants related to longevity, with 18 of them encompassing the exceptional longevity allele • Analysis reveals 58 trait‐associated variants compared to the NHGRI GWAS catalog, with 11 of them being homozygous for the risk allele • In addition, 25 novel variants within candidate genes for aging and longevity are discovered, along with seven longevity‐associated variants in the sample • Gene expression patterns associated with different T cell subsets: Tγδ cells (Vδ1 and Vδ2) and their functional subsets using markers defining Tαβ cells (CD27 and CD45RA) • DNA repair and damage response pathways • Clonal hematopoiesis as a crucial player for healthy aging and protection from cardiovascular events • Marked increase in cytotoxic CD4 T cells (CD4 cytotoxic T lymphocytes [CTLs]) as a signature of supercentenarians

Epigenetics	Telomere length and maintenance: telomeres are protective caps at the ends of chromosomes that shorten with each cell division and are associated with cellular aging [80, 81]	• Relative telomere length (RTL): it is a measure of telomere length compared to a reference sample • Predicted telomere length
	Epigenetic modifications: [82]	• Association of epigenetic age with mortality rates, association of epigenetic age with cardiovascular disease (CHD) risk factors
	DNA methylation age (DNAm Age) (epigenetic clock): it is a DNA methylation−based estimator of chronological age or mortality risk integrated into epidemiologic studies. They are used to measure the age of tissues and cells and have been linked to various pathologies, health states, lifestyle, mental state, and environmental factors [1, 30, 67]	• The Bekaert clock was used as a blood‐based age prediction model • Pan‐tissue clock • Skin and blood clock • DNAm PhenoAge clock • Hannum clock • Thong clock • Garali MQR clock • Garali GBR clock
	MicroRNAs: are small noncoding RNAs that regulate gene expression posttranscriptionally have been implicated in aging and age‐related diseases [80, 90]	• mir‐146a‐5p • mir‐126‐3p • mir‐21‐5p
	Blood mononuclear cells (PBMCs) methylation data [70, 120]	Dependent on blood cell counts and immune system age
	Extrinsic epigenetic age acceleration (EEAA) [120]	Independent of blood cell counts

Quantitative analysis and risk assessment	Polygenic risk scores (PRSs): PRSs estimate an individual’s genetic liability to a trait or disease based on their genotype profile and relevant genomewide association study (GWAS) data [110]	Exceptionally long‐lived individuals have a higher genetic risk for exceptional longevity but did not significantly differ in polygenic risk for most cardiovascular health traits
	Quantitative trait: are phenotypic traits determined by many genes of small effect influenced by the environment [121]	There is strong evidence that longevity is a heritable quantitative genetic trait, showing a significant survival advantage for individuals with the top 10% surviving relatives

Microbiome	Microbial diversity and stability in extreme longevity [87, 122].	High species diversity and stability are linked to longevity, supporting a resilient gut microbiome
	Antioxidant and detoxification mechanisms supporting extreme longevity [122, 123]	Enhanced antioxidant activity (via *Lactobacillus* and L‐ascorbic acid) and xenobiotic degradation protect against oxidative stress and environmental toxins
	Youth‐like microbiome features in supercentenarians [87].	Youth‐like gut signatures, including *Bacteroides* dominance and increased species evenness, contribute to microbiome stability and longevity
	Antibiotic resistance and health risks in extreme longevity [124]	Age‐related accumulation of antibiotic resistance genes, especially multidrug efflux pumps, poses health risks in extreme old age

#### 3.2.13. Ecological Theme

Understanding the role of environmental factors in aging is crucial for promoting healthy aging and reducing the burden of age‐related diseases. Two studies in our scoping review assessed the environmental factors. The first study investigated the environmental factors influencing supercentenarians by measuring toxic and essential trace metals, cadmium (Cd), lead (Pb), iron (Fe), and selenium (Se), in the fingernails of the participants. In addition, soil and rice samples were analyzed to understand environmental influences on these trace elements. The concentrations of Cd, Pb, and Se were determined, and the limits of detection (LODs) were 0.010 μg L^−1^ for Cd, 0.010 μg L^−1^ for Pb, 20 μg L^−1^ for Fe, and 0.050 μg L^−1^ for Se [88]. The second study focused on other environmental factors, including the quality of the drinking water supply, occupational exposure, passive smoking exposure, and the types of cooking and heating fuels used by the participants [94].

## 4. Discussion

This scoping review systematically mapped and synthesized the existing literature on supercentenarians and semisupercentenarians to identify key themes, methodologies, and findings that can set the foundation for the development of a Delphi survey that aims to collect experts’ consensus on the constituents of a global unified protocol for future supercentenarian cohort studies. Based on our review, we propose a comprehensive framework organized into three primary components: cohort setting, measuring contributing factors, and full assessment. This framework is designed to capture the multifaceted dimensions of exceptional longevity and address notable gaps in current research (Table 2). In addition to summarizing the current evidence, the academic value of this review lies in providing a structured, protocol‐oriented framework that can support harmonized data collection, improve comparability across settings, and strengthen future pooled analyses in a field where individual studies are often limited by the rarity of verified extreme‐age survivors.

**TABLE 2:** Framework can inform the development of a protocol for a global supercentenarian cohort study.

**Cohort setting**

Age validation
Brief screening (to define inclusion and exclusion criteria)
Physical health
Cognitive health
Mental health
Nutritional status
Sensory health
Functional status

**Measuring Contributing Factors**

Demographics
Social dimensions
Social support and engagement
Past engaged lifestyle
Social resources
Religiosity/spirituality
Lifestyle
Smoking
Alcohol consumption
Physical activity
Caffeine intake
Sexual life
Nutrition and diet
Sleep
Dental habits
Personality trait
Self‐esteem
Locus of control
Emotional intelligence
Social dominance orientation
Risk‐taking propensity
Openness, conscientiousness, extraversion, agreeableness, and neuroticism
Historical stress
Natural environment
Heavy metals exposure
Organochlorine pesticides exposure
Endocrine disruptors exposure
Air pollution exposure
Access to green spaces
Noise pollution exposure

**Full assessment**

Health conditions and past medical history
Cardiovascular health
Cerebrovascular history
Respiratory condition
Kidney condition
Cancer
Git conditions
Diabetes mellitus or high blood sugar
Infectious diseases
Reproductive history
Neurogenetic disorders
Vision or hearing problems
Disability status
Medical history: hospitalizations and ER admissions
Medication
Current health service use
Independence/self‐ability
Functional status
Fatigue
Pain
Frailty
Cognition
Mental health
Anxiety
Depression
Mental well‐being
Coping
Psychological strength
Loneliness
Quality of life
Clinical examination
Anthropometric measurements
Physical examination
Ultrasonography
Dental examination
Gynecological check‐up
Hematology and biochemistry
Genetics dimensions
Biomarkers of Aging

Exceptional longevity should be interpreted as a multilevel, life‐course phenomenon, not a single‐domain endpoint. Survival into ages 105+ and 110+ reflects decades of social and biological selection, so observed profiles among the oldest‐old are shaped by demographic survival selection (frailty/mortality filtering) and by the historical contexts of their birth cohorts [125, 126]. These selection dynamics have direct implications for molecular inference: longevity genetic signals can be altered by population structure and cohort heterogeneity and can be biased by mortality selection and nonrepresentative participation [127–129]. At the same time, the concept of longevity itself requires careful interpretation. Longevity, or lifespan, refers to the duration of survival, but it does not necessarily capture the quality of those added years. For this reason, exceptional survival should not automatically be equated with successful aging. A sociophilosophical perspective on the human lifespan suggests that living longer is not inherently synonymous with living well, and that longevity should be interpreted alongside autonomy, dignity, function, social connectedness, and QoL. Accordingly, genetic findings in supercentenarians and semisupercentenarians should be interpreted alongside harmonized demographic information (birth cohort, migration/ancestry, and validation procedures) and with careful attention to age‐ascertainment bias and verification standards that underpin credible extreme‐age phenotypes [127, 130].

In the cohort setting phase, the initial steps involve rigorous age validation and brief screening to define inclusion and exclusion criteria. A universal age validation technique that is applicable across diverse populations is essential to ensure consistency and accuracy. This requires developing robust strategies to resolve discrepancies in historical records, managing undocumented cohorts, and addressing complications such as name changes (e.g., due to marriage) and citizenship alterations. Standardized protocols for cross‐referencing multiple data sources, including civil registries, census records, and family testimonials, are vital for enhancing the reliability of age validation in global studies of supercentenarians. Foundational work on validated extreme‐longevity databases further emphasizes that unbiased inference at ages 110+ depends on meticulous validation procedures and explicit attention to ascertainment bias [130]. This is particularly important in global research, where documentation systems vary substantially across countries and where age misreporting can introduce major bias if not addressed systematically.

The measuring contributing factors component of the framework was informed by several key themes identified in the literature. These include demographics, social dimensions, lifestyle factors, personality traits, ecological dimensions, medical history, medication use, and healthcare service utilization. Notably, while historical stress was mentioned in only one study, its inclusion is critical for examining how supercentenarians navigated significant stressors, such as wars, economic crises, and societal changes. In addition, although environmental factors are crucial for understanding aging and longevity, only two studies addressed this theme. This gap is consequential because the “nongenetic” environment is not a single exposure but a cumulative profile across the life course (i.e., an exposome), which is socially patterned and can shape both disease risk and biological aging markers [131–133]. Therefore, expanding measurement of environmental and contextual domains (e.g., air pollution, noise, green space access, endocrine disruptors, organochlorine pesticides, and heavy metals) is not only descriptive but also analytically necessary to interpret biological signals and to reduce confounding driven by unequal exposure distributions [131–134].

A broader socioecological perspective further strengthens this point. Recent work has highlighted the survival probability of becoming a centenarian as a useful population‐level lens for understanding longevity, emphasizing that exceptional survival is shaped not only by individual biology and behavior but also by structural conditions that accumulate across the life course [135, 136]. In this view, longevity reflects the interaction between individuals and the wider social, economic, and environmental systems in which they age. Country‐level studies have shown that socioeconomic indicators such as national income, health expenditure, sanitation, and broader living‐standard proxies are associated with survival probability to age 100 [136]. Other work has shown that macrolevel social conditions, including gender inequality and women’s political representation, may also be associated with the survival probability of becoming a centenarian [137]. These findings support the inclusion of contextual socioecological variables in future global protocols, as they may help explain variation in exceptional longevity beyond individual‐level characteristics alone. These broader socioecological conditions may also shape how individuals experience historical stressors and environmental exposures across the life course, further reinforcing the importance of including both historical stress and environmental factors in the proposed framework.

The full assessment phase involves detailed evaluations that provide a holistic view of the health and well‐being of participants. This includes in‐depth assessments of health status, cognitive function, mental health, independence, QoL, and clinical parameters. Detailed clinical examinations such as anthropometric measurements, physical assessments, ultrasonography, dental examinations, and hematology and biochemistry analyses are critical for identifying biomarkers and understanding the underlying mechanisms of aging. Importantly, evaluating genetic dimensions may reveal survival‐associated variants, but such findings require interpretation in light of demographic structure (cohort specificity, ancestry/population structure, and selection) rather than as context‐free determinants [127, 128, 138, 139]. Similarly, epigenetic and other biomarker‐of‐aging measures are shaped by exposures and behaviors, and systematic evidence indicates that epigenetic aging measures covary with lifestyle/health factors and with broader social and environmental correlates [140–143]. Consequently, the value of a Global Supercentenarian Cohort Study is maximized when biological profiling is paired with high‐quality harmonized demographic, lifestyle, and socioecological data, enabling integrated models rather than parallel single‐domain analyses [132, 144].

At the same time, this cohort‐based framework should be understood alongside other available approaches to the study of longevity. Population‐demographic approaches, including analyses based on mortality databases and validated extreme‐longevity datasets, can help estimate survival probabilities and characterize mortality patterns at advanced ages. Ecological and country‐level analyses can generate hypotheses about how macrolevel social and economic conditions shape the production of centenarians [136, 137]. Registry‐based and administrative data linkage approaches may also help reconstruct life‐course exposures, health service use, and survival trajectories where such systems are available. However, while these approaches are valuable, they often lack the depth of harmonized phenotyping needed to connect demographic outcomes with cognitive, psychosocial, clinical, and biological profiles at the individual level. For this reason, a Global Supercentenarian Cohort Study offers an important complementary approach by integrating these domains within a single coordinated design.

A key limitation of the current evidence base is that research on supercentenarians and semisupercentenarians, although highly promising, remains constrained by the extreme rarity of individuals who reach these ages. Consequently, only a small number of studies have analyzed data from verified supercentenarians or semisupercentenarians, and much of the literature mapped in this scoping review is derived from centenarian cohorts, with only a limited subset including participants aged 105+ or 110+. While this reflects an unavoidable demographic reality, it limits comparability across studies and reduces the extent to which findings from centenarians can be extrapolated to the oldest‐old. This limitation also underscores why integrated approaches are essential: at extreme ages, cohort heterogeneity and survival selection can reshape the observed distribution of genetic, clinical, and biomarker measures, and study participation processes can further bias inference [125, 127, 129, 139]. It also highlights the conceptual limitation of longevity research more broadly: survival to extreme age is an informative outcome, but it is only one dimension of late‐life experience and should be interpreted alongside function, resilience, well‐being, and social context. This further reinforces the importance of a unified global protocol, particularly for age validation, eligibility screening, and harmonized measurement, so that data collection is consistent across settings and future studies can be more readily pooled, compared, and translated into stronger, more generalizable conclusions [130, 145].

Future research should build on this framework through several complementary strategies. First, studies should adopt a multilevel approach that combines individual‐level phenotyping with country‐ or region‐level socioecological indicators, allowing researchers to examine how structural context shapes exceptional survival [135–137]. Second, future cohorts should include prospective or repeated‐wave follow‐up wherever feasible, so that trajectories in function, cognition, frailty, and QoL can be studied rather than relying only on cross‐sectional assessments. Third, linkage to registries or administrative health records may improve the capture of hospitalizations, service use, medication histories, and survival outcomes in settings where infrastructure permits. Fourth, comparative designs, including contrasts between semisupercentenarians, centenarians, and younger‐old groups, may help disentangle what is specific to extreme‐age survival from what reflects broader aging processes. Finally, future work should continue to expand underdeveloped domains in the literature, particularly environmental exposures, historical stress, and socioecological context, so that exceptional longevity can be interpreted not only as a biological outcome but also as a product of cumulative life‐course opportunities, constraints, and adaptations [135–137].

Overall, the proposed framework not only identifies critical domains for assessment but also highlights important gaps in the literature, particularly in environmental exposures, historical stress, and broader socioecological influences, that should be addressed in future research. By integrating demographic verification and life‐course context (demography and socioecology) with behavioral exposures (lifestyle/exposome) and biological profiling (genetics, epigenetics, and clinical biomarkers), a Global Supercentenarian Cohort Study can reduce biased inference and better support mechanistic interpretation of exceptional longevity, including potential gene–environment correlation and effect modification pathways [127, 131–133, 138, 140, 144]. Such evidence can inform targeted interventions and policy initiatives to support healthy aging while acknowledging that observed signals in extreme‐age survivors reflect both biology and the structured opportunities and exposures that accumulate over the life course [126, 134, 146]. In this sense, the study of supercentenarians is valuable not only for understanding why a small number of people survive to extraordinary ages but also for clarifying how biological, social, and ecological systems interact across the lifespan to shape human longevity.

## Author Contributions

Conceptualizing: Omnia Abdelraheem, Wafa Abu El Kheir‐Mataria, and Sungsoo Chun.

Methodology: Omnia Abdelraheem, Wafa Abu El Kheir‐Mataria, and Sungsoo Chun.

Investigation: Omnia Abdelraheem, Wafa Abu El Kheir‐Mataria, and Sungsoo Chun.

Writing−original draft: Omnia Abdelraheem.

Writing−review and editing: Omnia Abdelraheem, Wafa Abu El Kheir‐Mataria, and Sungsoo Chun.

## Funding

This work was supported by the American University in Cairo (Grant no. SSE‐IGHHE‐S.C‐FY24‐RG‐2024‐Jan‐10‐15‐52‐32).

## Ethics Statement

The authors have nothing to report. This study was registered under Scoping Review Registration: PROSPERO CRD42024512298.

## Conflicts of Interest

The authors declare no conflicts of interest.

## Supporting Information

Appendix A: Detailed search strategy used across all databases (Scopus, PubMed, ProQuest, PsycINFO, and Cochrane), including full search queries, applied filters, and the number of retrieved records.

Appendix B: Overview of centenarian and supercentenarian studies conducted across different geographical locations, summarizing extracted themes, subthemes, and methodological characteristics.

## Supporting information


**Supporting Information** Additional supporting information can be found online in the Supporting Information section.

## Data Availability

Data sharing is not applicable to this article as no datasets were generated or analyzed during the current study.
