# Supplementary material for: Genetic, Socioecological, and Health Research on Extreme Longevity in Semisupercentenarians and Supercentenarians: A Scoping Review
Source: J Aging Res. 2026 Apr 17;2026:1605361. doi: 10.1155/jare/1605361 (PMC13090574; doi:10.1155/jare/1605361)
Supplement: Supplementary file 1 — Supporting Information Additional supporting information can be found online in the Supporting Information section. [file JARE-2026-1605361-s001.docx]

# Supplementary material:

## **Appendix A: Search Strategy**

Detailed search strategies used across all databases (Scopus, PubMed, ProQuest, PsycINFO, Cochrane, etc.), including filters and results.

Table S1 The Full Search Strategy

| Database | Search Query | Filters | Results |
| --- | --- | --- | --- |
| Scopus Articles | TITLE-ABS-KEY ( *supercentenarian* OR semi*supercentenarian ) AND ( LIMIT-TO ( DOCTYPE , "ar" ) OR LIMIT-TO ( DOCTYPE , "no" ) OR LIMIT-TO ( DOCTYPE , "cp" ) OR LIMIT-TO ( DOCTYPE , "sh" ) OR LIMIT-TO ( DOCTYPE , "le" ) OR LIMIT-TO ( DOCTYPE , "ed" ) ) | We included Articles, Notes, conference papers, letters and editorials.  We excluded reviews and book chapters | 160 Documents |
| PubMed | "supercentenarian"[Title/Abstract] OR "semi*supercentenarian"[Title/Abstract] OR (("Centenarians"[MeSH Terms] OR "Centenarians"[All Fields] OR "centenarian"[All Fields]) AND "Centenarians"[MeSH Terms]) | No | 178 entries |
| Proquest:  Ending Truncation:  Search query: supercentenarian* OR semi*supercentenarian  Middle Truncation:  Search query: supercenten*arian OR semi*supercentenarian | (supercentenarian* OR semi*supercentenarian supercenten*arian OR semi*supercentenarian) NOT (at.exact("Literature Review" OR "Review") AND PEER(yes))  Databases   1. Coronavirus Research Database 2. ProQuest Dissertations & Theses Global 3. Publicly Available Content Database | Peer reviewed.  Exclude literature reviews and reviews | 310 |
| PsycINFO | *supercentenarian* OR semi*supercentenarian | No | 32 |
| Scopus Secondary literature “A secondary document is a document extracted from a Scopus reference list, but is not indexed by, or available in, Scopus. | TITLE-ABS-KEY ( *supercentenarian* OR semi*supercentenarian ) | no | 105 Documents |
| Scopus Patents | TITLE-ABS-KEY ( *supercentenarian* OR semi*supercentenarian ) | no | 37 patents |
| Cochrane Database of Systematic Reviews (CDSR) | (centenarian):ti,ab,kw OR (supercentenarian):ti,ab,kw AND (semi-supercentenarian):ti,ab,kw | no | 16 trials  5 reviews  1 protocol |

## **Appendix B: Centenarian and Supercentenarian Studies**

Overview of studies conducted in different geographical locations, including countries and specific regions, with extracted themes, subthemes, and methodologies.

Table S2 Centenarian studies form different geographical locations

| **No** | **Study** | **Extracted Themes/Subthemes and Methodologies** |
| --- | --- | --- |
| 1 | The China Hainan Centenarians Cohort Study (CHCCS)  [1] | **Sociodemographic**  **Functional capacity**  **Cognitive function**  **Mental health**  **Behaviors**  **Habitual diet**   - (11-item Semi-Quantitative Food Frequency Questionnaire)   **Sleep Quality**   - (Pittsburgh Sleep Quality Index - PQSI),   **Quality of life (EQ-5D)**  **Family information**  **Social support/relations**  **Environment**  **Economic status**  **Health service use**  **Reproductive history.**  **The medical or clinical examination:**  **Health conditions,**  **Anthropometric measurements,**  **Falls**  **General pain,**  **Physical function,**  **Physical examination,**   - ECG, - Ultrasonography - Dental examination.   **Biological specimens:**   - Blood analysis, - Saliva DNA, - Hair analysis for trace elements, - Feces examination - Gynecological check-up. |
| 2 | The Georgia Centenarian Study  [2] | - Demographic information - Family longevity - Social and environmental support - Personality - Stress and coping - Life satisfaction and morale - Health - Mental health - Nutrition and dietary patterns - Religiosity - Reminiscence - Intelligence and cognition |
| 3 | The Fordham Centenarian Study  [3] | - Demographic aspects - health - cognitive status - social network and living arrangements - well-being aspects - psychological strength |
| 5 | Italian Centenarians and Semi Supercentenarians Surveys  [4] | - Age validation as prerequisite; bias reduction in extreme-age counts - Sex differences at extreme ages (femininity ratios) - Cohort survival trends beyond age 100; shifting age-at-death to oldest-old - Geographic/territorial distribution and internal migration (birth vs last residence) - SSC Survey (≥105; 2009–2018) (Istat; IDL-aligned) - Ascertainment via POSAS municipal registers - Document-based age validation: birth + living-existence/death certificates (exclude unconfirmed residents) - Annual revalidation and follow-up until death - Record linkage with national death/census sources (e.g., CDS/DRPS/census) - Centenarians (1870–1912 cohorts) reconstructed using Vincent extinct-cohort method - Analyses: trends by cohort/sex (incl. femininity ratios) + quantile regression of age-at-death (months) |
| 6 | Chinese Longitudinal Healthy Longevity Survey  [5] | - Health conditions - Daily functioning - Self-perceptions of health status and quality of life - Life satisfaction - Mental attitude - Feelings about aging - Diet and nutrition - Use of medical services - Drinking and smoking habits - Physical activities - Reading habits - Television viewing - Religious activities - Tested for motor skills, memory, and visual functioning |
| 7 | The Sevilla and Castilla Y Leon Centenarian Study  [6] | - Sociodemographic - Health status - Health history - Habits: physical exercise, smoking, drinking, hygiene, and diet - Activities of daily living (bathing, dressing, toileting, transferring, continence, and feeding |
| 8 | The New England Centenarian Study  [7] | - Longitudinal cohort (established 1994); expanded from a local population base to broader recruitment - Ascertainment via registries and community/healthcare channels - Strict age validation using multiple independent documents (birth + early-life records); enhanced checks for 110+ - Repeated phenotyping and annual follow-up when possible; proxy and medical-record verification as needed - DNA collection and genetics/omics analyses in subsets (e.g., genome-wide and sequencing efforts) |
| 9 | The Study of Danish Centenarians  [8] | - **Sociodemographic characteristics,** - **Activities of daily living,** - **Living conditions,** - **Need of assistance from other people,** - **Former health and current diseases,** - **Current medication** - **Clinical examination:** - Dementia screening test, - Heart and lung auscultation neurological assessment - Height and weight electrocardiogram - Arm and ankle blood pressure - Assessment of hearing and vision capacity - A short physical performance test - Bio-impedance - Lung function test - Blood test. |
| 10 | Pt100 Oporto Centenarian Study  [9] | **Sociodemographic**  **economic information**  health:  diseases,  subjective health  **Cognition**  **functional capacity:**  daily and instrumental activities of daily living, fatigue)  **Nutritional status and eating/drinking habits,**  **Mental health:**  anxiety  depression  **well-being**  satisfaction with life  **personal resources:**  personality and coping strategies  **social network, and support**  family relations,  use of formal services  The majority of these dimensions were evaluated through the Portuguese version of well-known instruments, such as:   - the Mini Mental State Examination (MMSE; Folstein et al. 1975), - the Global Deterioration Scale (Reisberg et al. 1982), - the Older Americans Resources and Services Multidimensional Functional Assessment Questionnaire (OARS, Fillenbaum and Smyer 1981), - the Lubben Social Network Scale (Lubben, et al. 2006), - the Valuation of Life Scale (Lawton et al. 2001), - the Brief Geriatric Anxiety Inventory (Byrne and Pachana 2011) - the Geriatric Depression Scale (Yesavage et al. 1983) to name a few. |
| 11 | Moscow Centenarian Study  [10] | **Frailty**: comprehensive geriatric assessment, including examination by the neurologist, an assessment of cognitive status, psychogeriatric consultation with the assessment of self-perceptions of aging and depression assessment by using the geriatric depression scale, neuropsychological testing using the Montreal Cognitive Assessment (MoCA), verbal recognition memory 12 word test, clock-drawing test.  **Relative telomere length in genomic DNA** and telomerase activity in monocytes will be assessed by real-time PCR.  **Vascular aging** will be measured by intima-media thickness measurement and plaque presence determination by ultrasonography in both left and right common carotid arteries. Arterial stiffness will be assess by aortic pulse wave velocity measuring by SphygmoCor. |
| 12 | Sydney Centenarian Study  [11] | - Medical history - Family medical history - Cognition, medications - Mental health - Subjective memory complaints - Falls history - Diet - Physical activity - Social integration - Social cognition |
| 13 | Tokyo Centenarian Study  [12] | Biomedical characteristics of centenarians,  Lifestyle habits,  Cognitive factors:   - Personality and - Approach to life,   Genetic features. |
| 14 | Second Heidelberg Centenarian Study  [13] | - Daily challenges - Person characteristics - Activities - Social network - Quality of life |
| 15 | Life At Age 100: An International Research Agenda for Centenarian Studies.  [14] | Group of studies including Fordham Centenarian Study and Second Heidelberg Centenarian Study.   - Predictors of anxiety in centenarians: Health, economic factors, and loneliness. |
| 16 | Hong Kong Centenarian Study  [15] | Semi-structured open-ended questions to assess psychological well-being of the participants:   - Social network contact and family support - Life events - Life attitude - Hope - Happiness - Congruence - Personality - Motto and suggestions to others regarding living a long life.   Chinese Longitudinal Healthy Longevity Survey  Elderly Health Center questionnaire Data collected included:   - family structure, - general functioning, - activities of daily living, - physical health - cognitive function. - Relevant demographic - socioeconomic   environmental data |
| 17 | Australian Centenarian Study  [16] | **Personality trait**   - NEO Five Factory Inventory (NEO-FFI) - Connor–Davidson Resilience Scale (CD-RISC)   Life Orientation Test Revised (LOT-R) |
| 18 | Cuba's National Centenarian Study  [17] | The Cuban study had three main goals;  1-Evaluate:   - physical - mental health status, - social conditions   2-Propose strategies to solve problems identified in the course of the study.  3-Establish healthy aging indicators that can be used to plan and conduct population-wide health interventions in the future. |
| 19 | Japanese Centenarians Study  [18] | - Adls - Cognitive status - Psychosocial status. |
| 20 | Okinawa Centenarians Study  [19] | - The diets, - Exercise habits, - Genetics, - Psychological - Spiritual practices, - Social and behavioral patterns - Among the oldest-old and count more than 3,000 participants |
| 21 | The Finnish Centenarians Study  [20] | The health interview: a wide range of diseases, hospitalizations, use of medication, and activities of daily living (ADL), of which the basic ADL comprised three questions: (1) assistance in having meals, (2)assistance in standing up, and (3) assistance in getting dressed. A final question on the need of daily assistance was also incorporated.  -The objective clinical examination included an ECG and blood samples |
| 22 | The Swedish Centenarians Study  [21] | **Sociological data**:   - marital status - type of housing - socioeconomic status - satisfaction with their professional life - education - social network (quantitative and qualitative, contact frequencies) - feelings of loneliness according to  UCLA-scale - formal/informal support.   **The medical examination:**   - Medical history, - questions concerning dietary and smoking habits and alcohol consumption, - general bedside examination, measurements of blood pressure in arms and legs, - ECG, - Mid arm circumference, - triceps and subscapular skinfolds, body composition using an impedance technique - bedside assessment of hearing capacity, - near visual acuity, - ADL function according to ADL index of Katz - general mental function according to the Berger scale   **Blood   samples for examinations of:**  B-hemoglobin,  leukocyte count,  S-iron,  S-total iron binding capacity, B-folates,  S-cobalamin,  S-creatinine,  S-sodium,  S-potassium,  S-calcium,  S-ASAT,  S-ALAT,   S-GT,  S-alkaline phophatase, S-HDL-cholesterol, P-a2-microglobulin,  P-prealbumin,  P-albumin,  P-a'- antitrypsin,  P-orosomucoid,  P-haptoglobin,  CRP, P-ceruloplasmin,  P-IgG,  P-IgA,  P-IgM,  P-C3,  P-C4.  **Psychological assessment**   - word list from the CVB, 1/2-scale - Digit Span Backwards  and  Forwards - Memory for designs - the five-objects  test, as used in clinical practice - learning- retention test - simple  reaction  time. - observation of behavior - quality of life and personality rating   **A psychiatric   examination**  **A neuropathological examination** |
| 23 | Centenari A Trieste’ (Cat), A Study of The Health Status of Centenarians in a Small Defined Area of Italy.  [22] | -Clinical visit to collect data on present and past pathologies and on daily living activities  -Neuropsychological assessment during which the patients were tested using an ad hoc battery on cognitive and mood domains. |
| 24 | The 100-Plus Study.  [23] | - General: ICF, broof of age, childhood living environment, disease history, liestyle - Cognitive: memory, attention, language, Visuo-spatial functioning, executive functioning - Depression, ADL, sleep, lifestyle, geriatric impairments - Vision, hearing, mobility - Blood sample |
| 25 | The Galician Longevity Study (2001).  [7] | In 2001, a population-based study from the province of Galicia identified all 99-year-and-older  persons in a central registry (Rabunal-Rey  et al. 2012).  The questionnaire included   - diseases - ADL functioning by the Barthel - Index   The objective examination included blood pressure measurement,   - blood sampling, and - ECG. |
| 26 | The Korean Centenarian Study (2005).  [24] | **ADL dependency**  **Functional disabilities**  **Health states,** where health state would be defined by questions on having had eight specific diseases common in old age. |
| 27 | The Spanish Centenarian Study (2011–2013).  [7] | **The health interview focused on:**  **Cardiovascular morbidity.**  **Clinical examination:**  ECG  Echocardiography. |

# References:

[1] Y. He *et al.*, “Cohort Profile: The China Hainan Centenarian Cohort Study (CHCCS),” *Int. J. Epidemiol.*, vol. 47, no. 3, pp. 694–695h, Jun. 2018, doi: 10.1093/ije/dyy017.

[2] L. W. Poon *et al.*, “The Georgia Centenarian Study,” *Int. J. Aging Hum. Dev.*, vol. 34, no. 1, pp. 1–17, Jan. 1992, doi: 10.2190/8M7H-CJL7-6K5T-UMFV.

[3] D. S. Jopp and S. Hicks, “Fordham Centenarian Study,” in *Encyclopedia of Geropsychology*, Springer, Singapore, 2016, pp. 1–9. doi: 10.1007/978-981-287-080-3_183-1.

[4] G. Caselli, M. Battaglini, and G. Capacci, “Italian Centenarians and Semi-supercentenarians Surveys,” in *Encyclopedia of Gerontology and Population Aging*, Springer, Cham, 2021, pp. 2790–2799. doi: 10.1007/978-3-030-22009-9_1000.

[5] “Chinese Longitudinal Healthy Longevity Survey (CLHLS), 1998-2002.” Accessed: Feb. 14, 2026. [Online]. Available: https://www.icpsr.umich.edu/web/NACDA/studies/3891

[6] J. M. García-González and A. del Rey, “Research on Individuals Aged One Hundred and Over: Protocol from the Sevilla and Castilla y León Centenarian Studies,” *Int. J. Qual. Methods*, vol. 20, p. 16094069211031125, Jan. 2021, doi: 10.1177/16094069211031125.

[7] S. H. Rasmussen and K. Andersen-Ranberg, “Health in Centenarians,” in *Encyclopedia of Geropsychology*, Springer, Singapore, 2016, pp. 1–13. doi: 10.1007/978-981-287-080-3_78-1.

[8] K. Andersen-Ranberg and B. Jeune, “The Danish Longitudinal Centenarian Study,” in *Longer Life and Healthy Aging*, Z. Yi, E. M. Crimmins, Y. Carrière, and J.-M. Robine, Eds., Dordrecht: Springer Netherlands, 2006, pp. 151–172. doi: 10.1007/1-4020-4032-6_11.

[9] O. Ribeiro, L. Araújo, L. Teixeira, D. Brandão, N. Duarte, and C. Paúl, “Oporto Centenarian Study,” in *Encyclopedia of Geropsychology*, Springer, Singapore, 2015, pp. 1–7. doi: 10.1007/978-981-287-080-3_141-1.

[10] Pirogov Russian National Research Medical University, “A Model for Healthy Aging: Moscow Centenarians (AGE-100),” clinicaltrials.gov, Clinical trial registration NCT02876809, Mar. 2021. Accessed: Feb. 14, 2026. [Online]. Available: https://clinicaltrials.gov/study/NCT02876809

[11] “Sydney Centenarian Study (SCS) | CHeBA - UNSW Sydney,” UNSW Sites. Accessed: Feb. 14, 2026. [Online]. Available: https://www.unsw.edu.au/cheba/research-and-impact/research-projects/sydney-centenarian-study-scs

[12] Center for Supercentenarian Research, Keio University School of Medicine. “The Tokyo Centenarian Study (TCS).” Keio University School of Medicine – Center for Supercentenarian Research [website in Japanese/English]. Accessed Feb. 14, 2026. Available at: https://www.keio-centenarian.com/english/tcs

[13] C. Rott, D. S. Jopp, and K. Boerner, “Heidelberg Centenarian Studies,” in *Encyclopedia of Geropsychology*, Springer, Singapore, 2017, pp. 1042–1049. doi: 10.1007/978-981-287-082-7_63.

[14] D. S. Jopp, K. Boerner, O. Ribeiro, and C. Rott, “Life at Age 100: An International Research Agenda for Centenarian Studies,” *J. Aging Soc. Policy*, vol. 28, no. 3, pp. 133–147, Jul. 2016, doi: 10.1080/08959420.2016.1161693.

[15] S. Cheung *et al.*, “Healthy longevity and health care service needs: a pilot study of the centenarians in Hong Kong,” vol. 7, no. 1, 2012.

[16] J. Law, R. L. Richmond, and F. Kay-Lambkin, “The contribution of personality to longevity: Findings from the Australian Centenarian Study,” *Arch. Gerontol. Geriatr.*, vol. 59, no. 3, pp. 528–535, Nov. 2014, doi: 10.1016/j.archger.2014.06.007.

[17] “Counting to 100: A First Look at Cuba’s National Centenarian Study.” Accessed: Feb. 14, 2026. [Online]. Available: https://mediccreview.org/counting-to-100-a-first-look-at-cubas-national-centenarian-study/

[18] A. Ozaki, M. Uchiyama, H. Tagaya, T. Ohida, and R. Ogihara, “The Japanese Centenarian Study: Autonomy Was Associated with Health Practices as Well as Physical Status,” *J. Am. Geriatr. Soc.*, vol. 55, no. 1, pp. 95–101, Jan. 2007, doi: 10.1111/j.1532-5415.2006.01019.x.

[19] “ORCLS – Okinawa Research Center for Longevity Sciences.” Accessed: Feb. 14, 2026. [Online]. Available: https://www.orcls.org/index.html

[20] J. Louhija, *Finnish centenarians: a clinical epidemiological study*. Helsinki ; J. Louhija, 1994.

[21] S.-M. Samuelsson *et al.*, “The Swedish Centenarian Study: A Multidisciplinary Study of Five Consecutive Cohorts at the Age of 100,” *Int. J. Aging Hum. Dev.*, vol. 45, no. 3, pp. 223–253, Oct. 1997, doi: 10.2190/XKG9-YP7Y-QJTK-BGPG.

[22] “Cohort profile: ‘Centenari a Trieste’ (CaT), a study of the health status of centenarians in a small defined area of Italy | BMJ Open.” Accessed: Feb. 14, 2026. [Online]. Available: https://bmjopen.bmj.com/content/8/2/e019250.long

[23] “The 100-plus Study of cognitively healthy centenarians: rationale, design and cohort description on JSTOR.” Accessed: Feb. 14, 2026. [Online]. Available: https://www-jstor-org.libproxy.aucegypt.edu/stable/45217267?seq=1

[24] S. C. Park, “THE SECRET OF LONGEVITY OF KOREAN CENTENARIANS,” *Innov. Aging*, vol. 1, no. Suppl 1, p. 1074, Jun. 2017, doi: 10.1093/geroni/igx004.3934.
